# A Stochastic Spatiotemporal Model of Rat Ventricular Myocyte Calcium Dynamics Demonstrated Necessary Features for Calcium Wave Propagation

**DOI:** 10.3390/membranes11120989

**Published:** 2021-12-18

**Authors:** Tuan Minh Hoang-Trong, Aman Ullah, William Jonathan Lederer, Mohsin Saleet Jafri

**Affiliations:** 1 School of Systems Biology, Krasnow Institute for Advanced Study, George Mason University, Fairfax, VA 22030, USA; hoangtrongminhtuan@gmail.com (T.M.H.-T.); aullah3@gmu.edu (A.U.); 2 Center for Biomedical Engineering and Technology, University of Maryland School of Medicine, Baltimore, MD 21201, USA; JLederer@som.umaryland.edu

**Keywords:** arrhythmia, calcium waves, heart, computational model

## Abstract

Calcium (Ca^2+^) plays a central role in the excitation and contraction of cardiac myocytes. Experiments have indicated that calcium release is stochastic and regulated locally suggesting the possibility of spatially heterogeneous calcium levels in the cells. This spatial heterogeneity might be important in mediating different signaling pathways. During more than 50 years of computational cell biology, the computational models have been advanced to incorporate more ionic currents, going from deterministic models to stochastic models. While periodic increases in cytoplasmic Ca^2+^ concentration drive cardiac contraction, aberrant Ca^2+^ release can underly cardiac arrhythmia. However, the study of the spatial role of calcium ions has been limited due to the computational expense of using a three-dimensional stochastic computational model. In this paper, we introduce a three-dimensional stochastic computational model for rat ventricular myocytes at the whole-cell level that incorporate detailed calcium dynamics, with (1) non-uniform release site placement, (2) non-uniform membrane ionic currents and membrane buffers, (3) stochastic calcium-leak dynamics and (4) non-junctional or rogue ryanodine receptors. The model simulates spark-induced spark activation and spark-induced Ca^2+^ wave initiation and propagation that occur under conditions of calcium overload at the closed-cell condition, but not when Ca^2+^ levels are normal. This is considered important since the presence of Ca^2+^ waves contribute to the activation of arrhythmogenic currents.

## 1. Introduction

Ca^2+^ is released from the sarcoplasmic reticulum (SR) intracellular Ca^2+^ store in the form of Ca^2+^ sparks at discrete microdomains known as calcium release sites of size ~300 nm in width and ~12 nm in height [1]. The large size of the ventricular myocyte (~120 µm in length, ~20 µm in width and ~15 µm in depth) allows calcium signals to propagate as Ca^2+^ waves if the conditions are right. The links between calcium sparks and calcium waves have been documented before and has been implicated in the origin of cardiac arrhythmias [2,3,4]. During Ca^2+^ alternans, a wave-like Ca^2+^ release was observed during systole in ventricular myocytes by triggering the cells with a small stimulus [5,6]. However, the mechanism and conditions under which calcium sparks can form a propagating calcium wave is still under investigation. The relation between Ca^2+^ sparks and Ca^2+^ waves is complicated by the cell structure, asymmetric spatial distribution of RyR2 clusters, anisotropic diffusion of Ca^2+^, Ca^2+^ sensitivity of CRUs, etc. Although fluctuations in RyR2 gating have been linked to the development of calcium waves in pathological situations, the links between RyR2 activity and propagation of calcium waves are not well understood. Ca^2+^ wave generation in myocytes is linked to RyR2 gating and sarcoplasmic reticulum Ca^2+^ overload [7]. Ca^2+^ waves in rat ventricular myocytes have been observed during diastole under Ca^2+^ overload conditions [8,9,10].

How the nature and properties of calcium sparks contribute to the generation of Ca^2+^ waves and arrhythmias has raised intriguing issues in the field since Ca^2+^ sparks were discovered [1,11,12,13,14]. Among them, there is a major unresolved problem between the full-width at half-maximal concentration (FWHM) of experimentally recorded Ca^2+^ sparks (which is about 1.8–2.2 µm) and the pseudo-line scan generated Ca^2+^ spark from computational modeling (which is about 1.1–1.6 µm) [15,16,17,18,19,20,21]. Izu and co-workers developed a 3D model with spherical geometry using FASCIMILE (AEA Technologies, Harwell, UK) for studying Ca^2+^ sparks. In their model, in order to reproduce FWHM ~2 µm, they created a supercluster with 4 CRUs at 0.4 µm apart and a very large release of Ca^2+^ was assumed, i.e., from 2 pA to 5–10 pA for each CRU [17]. A recent 3D model developed using FASCIMILE by Kong and co-workers also produced a FWHM of 1.2 μm [22]. Such models are not supported by physiological measurements and anatomic characterizations of the CRUs.

Here, we have developed a more realistic model. The study of Ca^2+^-spark-induced Ca^2+^ wave started with simple deterministic models [4,23] or stochastic models using a simple representation of calcium release units. Keizer and co-workers, using a simple single-channel model of CRUs, with no buffers and very high Ca^2+^ sensitivity (K_Ca_ < 1.5 μM), suggested that the threshold for wave propagation is governed by a single, dimensionless parameter Dτ/d^2^, where τ is the mean time that a site is open, *D* is the diffusion constant of Ca^2+^ and the distance *d* between 2 sites. The wave is continuous when $D\tau /d^{2}\gg 1$, or saltatory if $D\tau /d^{2}\ll 1$ [24,25]. Izu and co-workers used their large CRU model in a 2D setting, with the fixed open time for Ca^2+^ release and a more realistic Ca^2+^ sensitivity (K_Ca_ = 15 μM), estimated P_o,trigger_ depending on the distance *d* and the rate and total mass of the source Ca^2+^ release [26]. Ramay and co-workers created a 3D model to study the Ca^2+^ wave, using a T-tubule of 200 nm as a diffusion barrier [27]. The model didn’t incorporate the sarcolemma and its ionic current. In 2010, a 2D model to study spark-induced spark was created by Rovetti and co-workers to form a network of 100 × 100 CRUs with each CRU occupying a domain of 5 × 5 grids [28]. However, their model wasn’t able to produce a proper spark model, i.e., the peak [Ca^2+^]_i_ < 10 μM, while the estimated level should be around 100 μM [1]. A recently developed 3D model for the rabbit ventricular myocyte by Nivala and co-workers [29] extended the previous model by Rovetti et al. [28] to 3D with 100 × 20 × 10 CRUs. In their model, they assumed (1) fast buffering, (2) the transmembrane voltage senses the calcium everywhere, (3) a simple Hill equation to describe the SERCA pump which does not capture true pump dynamics, (4) no flux into the subspace calcium and the calcium in the dyadic subspace is at equilibrium, and (5) Ca^2+^-dependent inactivation, however, the model cannot reproduce realistic Ca^2+^ sparks. The model described in this paper overcomes all these issues.

For atrial cells, due to the lack of the transverse T-tubule system or reduced T-tubule presence and altered properties, the physiological activation of the contraction process requires the propagation of Ca^2+^ in the form of Ca^2+^ waves from the sarcolemma to the interior of the cell [30,31]. Thul and co-workers developed a 3D model in which the stochastic nature of Ca^2+^ release was simply modeled using a threshold model in which a random variable is generated to determine whether Ca^2+^ is released or not [32].

At the whole-cell level, Shiferaw and colleagues [33] split the cells into subregions that each represented an individual sarcomere and combined them into 1D chains of these identical subregions to approximate the myocyte. Li and co-workers [34] developed the first detailed model by describing the cell as a cylinder with space step 200 nm, with cell volume 20.1 pL and modeling the release of calcium as a point source. Thus, they were unable to investigate the stochastic nature of calcium release which is important to the triggering of calcium waves [35].

We first introduce the 3D spatiotemporal model of the rat ventricular myocyte and then focus on the computational methodology used in model development. The major contribution of this study is two-fold: it investigates the probabilities and conditions for Ca^2+^ spark-induced sparks to occur, and the probabilities and conditions for spark-induced waves to occur. 

## 2. Materials and Methods

### 2.1. Model Development

**Figure 1:**
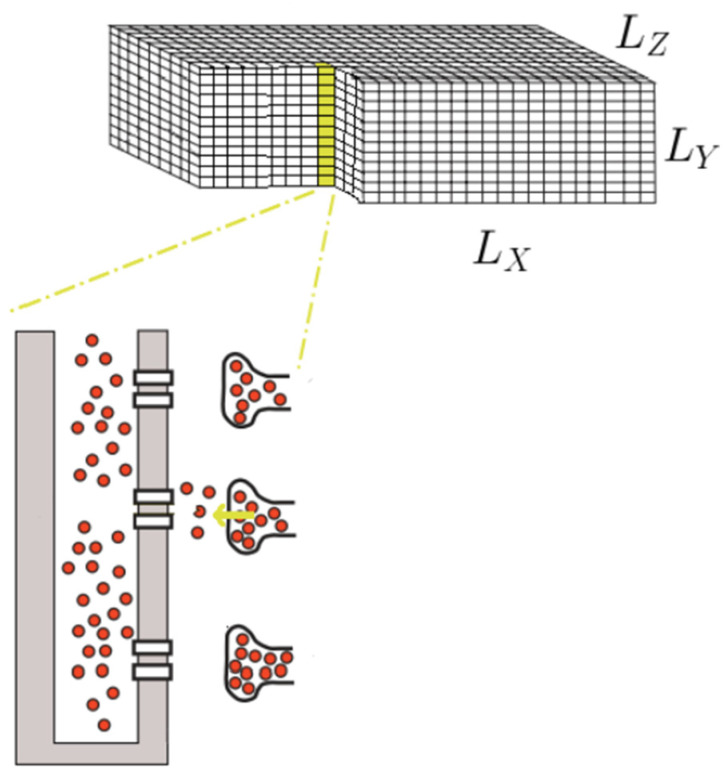
A schematic diagram of ventricular myocytes modeled as a rectangular solid.

Based on a previously published compartmental model for a rat ventricular myocyte, we created a spatial model [36]. The rat ventricular myocyte is represented as a rectangular solid with dimensions of 120 μm×20.8 μm×10 μm, yielding a total cell volume of 24.96 pL, which is consistent with the cell volume with 20,000 CRUs [1,37,38]. To capture the local effect of calcium release, the rectangular cell is divided into grid points of size 0.2 μm×0.2 μm×0.2 μm, yielding a total of 3,120,000 grid points, as shown in Figure 1.

The traditional electrophysiological approach of using total membrane capacitance to estimate the surface area and as an indirect index of cell volume is considered not accurate due to the variation in degree of membrane folding. Despite differences in cell volumes and capacitance-to-volume ratios between species, data from optical cell sections obtained with laser scanning confocal microscopy (LSCM) revealed a surprisingly constant membrane capacitance-to-cell volume ratio for each species over a wide range of cell volume using Sprague-Dawley rats (with volumes ranging from 23 pL to 63 pL) [38].The capacitance-volume ratio in rats is quite high (8.42 pF/pL), despite the fact that it varies with age (about 6.76 pF/pL in 3-month-old rats and 8.88 pF/pL in 6-month-old rats). In the Wistar rat (with cell volume 23.6 pL and capacitance 200 pF), a comparable number (8.43 pF/pL) was measured [39]. Other rat models, namely 6.25 pF/pL, substantially underestimate this [40,41].

### 2.2. Sarcolemma(SL) and T-Tubule Membrane

Given that the specific capacitance of cell membranes is consistent across cell types and species, $C_{\mathrm{sc}}\approx 1\mu F/{\mathrm{cm}}^{2}$ the total membrane surface area was given as $A_{m}=2.00\times 10^{-10}{\mathrm{cm}}^{2}\;$and the equivalent whole-cell membrane capacitance in the model is 200 pF. The surface membrane and the membrane in the T-tubular system are both part of the sarcolemmal membrane. Depending on the cell type, the thickness of a single cell’s plasma membrane is typically in the range of a few to tens of nanometers [42]; consequently, it can be easily fit into a single grid point of the size employed in the model. If we assume that the surface membrane at each grid point is 1.12 times the area of one side of the grid point, and using the total sarcolemma area above, it requires 445,000 grid points. The total number of grid points for the external surface membrane takes only 195,200 grid points, which means more than half of the membrane (i.e., 56%) belongs to the T-tubular system.

The fraction of total surface membrane area that forms T-tubules is known to be species-dependent. Early measurements revealed that it is approximately 33% in rats [43,44], with approximately 20–50% of the T-tubular membrane being part of the junctional complex with the junctional-SR [45]. Electron microscope measurements of cell capacitance after formamide-induced detubulation indicate a comparable value (32%) [46]. Recent optical measurements, however, revealed that the fraction of folded membrane forming the T-tubular system in rats is substantially higher (about 65%) [47,48]. The T-tubule fractions would be in the range of 50 to 65% based on the development stage-dependent volume-ratio data [37,49]. Thus, the value of 56% in the model is in the range. The discrepancy between the capacitance reduction and detubulation measurement can be explained by incomplete detubulation, structural distortions during the preparation of tissue for electron microscopy and even the smaller specific capacitance (0.56 µF/cm^2^) in the T-tubule due to high cholesterol level as suggested by [49].

Furthermore, new research showed that, despite the fact that T-tubules leave the surface membrane at Z-lines, only around 60% of the tubular volume occurs near the Z-line [47]. The number of grid points along the Z-line that include the T-tubule in our model is 151,000, which means the number of longitudinal grid points that constitute the branching T-tubules is 101,000 (40.6%). This is close to the value (40%) estimated experimentally in rats [47,50]. Due to the fact that the membrane folds not only in the transverse direction but also in the axial direction, we call it a transverse-axial tubular system (TATS or T-Ax), as suggested by [51] or sarcolemmal Z rete (ZRE) [47]. The TATS is also referred to as the sarcolemmal tubule network [52]. Several studies suggest that disruption or loss of the TATS is associated to a variety of cardiac diseases, and that understanding the link between them is critical for treatment development [53,54,55,56,57,58]. This emphasizes the importance of developing a spatiotemporal model for the ventricular myocyte that incorporates the detailed structure of the TATS to improve our understanding of its function.

In summary, the external sarcolemmal membrane spans the 6 surfaces of the rectangular solid. The TATS is composed of two parts. The T-tubular part is assumed to span along the *y*-axis of the cell, co-localizing at the calcium release site placements. The longitudinal part of the TATS is added to the network forming 40.6% of the membrane folding.

### 2.3. Calcium Release Site

The dynamics of calcium and calcium-calmodulin (CaCalm) complex at each release site is given by the equations:

(1) $$\frac{d{\left[\mathrm{Ca}\right]}_{\mathrm{ds}}^{\left(i\right)}}{\mathrm{dt}}=\frac{\left(J_{\mathrm{ryr}}^{\left(i\right)}-J_{\mathrm{efflux}}^{\left(i\right)}+J_{\mathrm{dhpr}}^{\left(i\right)}\right)}{\lambda_{\mathrm{ds}}}-2\frac{d{\left[\mathrm{CaCalm}\right]}^{\left(i\right)}}{\mathrm{dt}}-\frac{d{\left[\mathrm{CaSL}\right]}^{\left(i\right)}}{\mathrm{dt}}-\frac{d{\left[\mathrm{CaSR}\right]}^{\left(i\right)}}{\mathrm{dt}}$$

(2) $$\frac{d{\left[\mathrm{CaCalm}\right]}^{\left(i\right)}}{\mathrm{dt}}=k_{\mathrm{Calm}}^{+}{\left({\left[\mathrm{Ca}\right]}_{\mathrm{ds}}^{\left(i\right)}\right)}^{2}\left({\left[\mathrm{Calm}\right]}_{T}-{\left[\mathrm{CaCalm}\right]}_{\mathrm{ds}}^{\left(i\right)}\right)-k_{\mathrm{Calm}}^{-}{\left[\mathrm{CaCalm}\right]}_{\mathrm{ds}}^{\left(i\right)}$$

(3) $$\frac{d{\left[\mathrm{CaSL}\right]}^{\left(i\right)}}{\mathrm{dt}}=k_{\mathrm{SL}}^{+}\left({\left[\mathrm{Ca}\right]}_{\mathrm{ds}}^{\left(i\right)}\right)\left({\left[\mathrm{SL}\right]}_{T}-{\left[\mathrm{CaSL}\right]}_{\mathrm{ds}}^{\left(i\right)}\right)-k_{\mathrm{SL}}^{-}{\left[\mathrm{CaSL}\right]}_{\mathrm{ds}}^{\left(i\right)}$$

(4) $$\frac{d{\left[\mathrm{CaSR}\right]}^{\left(i\right)}}{\mathrm{dt}}=k_{\mathrm{SR}}^{+}\left({\left[\mathrm{Ca}\right]}_{\mathrm{ds}}^{\left(i\right)}\right)\left({\left[\mathrm{SR}\right]}_{T}-{\left[\mathrm{CaSR}\right]}_{\mathrm{ds}}^{\left(i\right)}\right)-k_{\mathrm{SR}}^{-}{\left[\mathrm{CaSR}\right]}_{\mathrm{ds}}^{\left(i\right)}$$

where i represents the index of the calcium release sites (i = 1…20,000). J_ryr_ is the flux of calcium release via RyR2 channels. J_efflux_ is the flux of calcium from the dyadic subspace (ds) into the cytosol. The volume ratio $\lambda_{\mathrm{ds}}=V_{\mathrm{ds}}/V_{\mathrm{myo}}$ was incorporated into the fluxes to address the volume differences between model compartments. The model used for this study is based on our published work where the reader can find all the details of the ionic currents and other details [36].

### 2.4. Spatial Placement of CRUs

**Figure 2:**
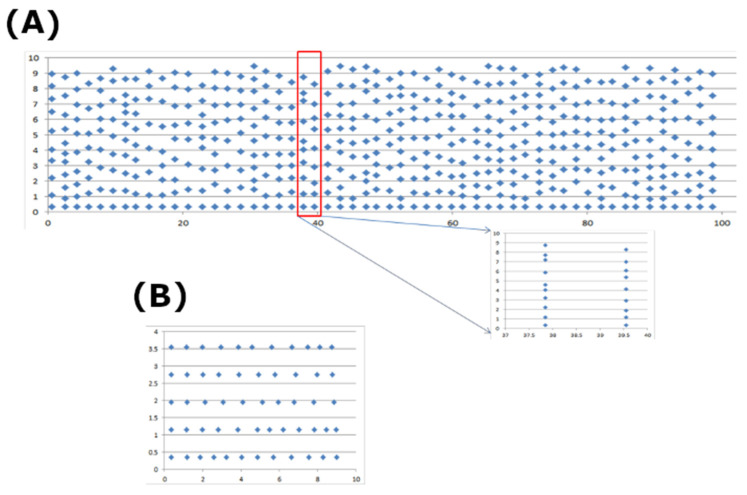
The placement of calcium release sites (**A**) at one Z-depth, and (**B**) at one Z-disc. The inset in (**A**) shows the CRUs on two T-tubules at two adjacent Z-discs. The distribution of inter-CRU distance is derived based on the experimental data.

Detubulation reduces the calcium current (I_Ca_) by 87% in L-type calcium channels, suggesting a smaller fraction on the surface membrane (Kawai, Hussain et al., 1999) [46]. This was incorporated in the model by using 13% of CRU on the surface membrane. There are two strategies for placing CRUs: (1) uniformly along each dimension at a given distance, or (2) non-uniformly based on the given distribution of nearest distance along the T-tubule. In the first case, a typical assumption is 1.8 µm along × and 0.8 µm along the transversal direction. In the second case, an algorithm was developed to place CRUs based on the distribution between adjacent CRUs along each T-tubule measured previously [59]. An example of the generated location of CRUs on a given depth value is shown in Figure 2.

### 2.5. Spatial Placement of Na^+^/Ca^2+^ Exchangers, SERCA Pump, SR and SL Buffers

The main pathway for Ca^2+^ extrusion is via the Na^+^/Ca^2+^ exchanger (NCX). With immunofluorescence and/or immunoelectron microscopy, the distribution of NCX was unclear, with both even distribution between external membrane surface and the T-tubules in rats and guinea pigs [60] and higher distribution in the T-tubular system in guinea pigs [61]. In another study for rat ventricular myocytes, NCX distributed largely in the T-tubule, yet NCX has not been observed in the dyad [62]. In a more recent study, by measuring the rise in extracellular calcium, ref. [63] showed that the detubulated cell has a smaller rise which suggested a higher distribution of NCX in T-tubules in rats, yet they could not quantify the change. Experiments using formamide-induced detubulation, ref. [64] estimated NCX to be 3–3.5-fold more concentrated in the T-tubules. However, in these studies, the decrease in cell surface area in detubulated cells is in the range 25–32%. Therefore, we focus on modeling NCX with 3 times higher in the T-tubules than in the external sarcolemma.

The transport of Ca^2+^ from the cytosol of the cardiomyocyte to the lumen of the sarcoplasmic reticulum (SR) is the major mechanism of removing Ca^2+^, therefore, it plays a major role in the contraction-relaxation cycle of the myocardium. There are different isoforms of SERCA pump that have been found in cardiac myocytes; with some being species-specific. In rat cardiac myocytes, SERCA2a is the major cardiac isoform while the level of SERCA2b is small. In mice, unlike SERCA2b which has a preferential localization around the T-tubules, SERCA2a is distributed transversely and longitudinally in the SR membrane [65,66]. Using immunostaining of rat ventricular myocytes with anti-SERCA2a primary antibody, it shows a uniform striated pattern where the brightest regions on the image are the Z-lines of the sarcomeres [16]. Smith and Keizer modeled this using a hypothetical function that is bell-shaped with the peak at the Z-line. The authors of [67] also found the presence of SERCA2a in the perinuclear region of the cardiac cell, yet the signal is weaker than that in the Z-lines. It is also noted that phospholamban (PLB) interact directly with SERCA2a to inhibit the uptake of Ca^2+^ back to the SR. Phosphorylation of PLB, which relives the inhibition of PLB on SERCA2a, can occur at two sites: Ser16 (PLB-16P) and Thr17 (PLB-17P), via cAMP-dependent kinases (PKA) and Ca^2+^/Calm-dependent kinase (CaMKII). Thus, the subcellular distribution of PLB-16P and PLB-17P can be of functional significance. The authors of [67] found that PLB-16B has a higher density at the Z-lines and perinuclear region; while PLB-17 is stronger at the Z-lines, in the intercalated disk region, and the external surface membrane. In essence, PLB-17 is found mainly in the region of importance to the EC-coupling. However, here we only focus on SERCA2a. The role of PLB will be a part of future studies.

In addition to calmodulin and troponin, SR, and SL also play an important role as Ca^2+^ buffers. The membranes near the Z-line have a greater Ca^2+^ buffering capacity than those close to the M-line [19]. Thus, in our model, 90% of the SR and SL buffers are assumed near the Z-line.

### 2.6. Diffusion of Ions

The diffusion of Ca^2+^ in aqueous solution of physiological ionic strength was estimated at about 700–800 μm^2^/s [68]. Kushmerick and Polodosky estimated that the diffusion coefficient in muscle is 50 times slower than in aqueous solution, i.e., D_Cafree_ = 14 μm^2^/s [69]. However, recent data suggested the diffusion in cytosol of free (unbuffered) Ca^2+^ is only 2–2.5× [16], with the value 400 μm^2^/s in smooth muscle cells and 223 μm^2^/s from *Xenopus laevis* oocytes [70,71]. Other studies used D_Cafree_ = 300 μm^2^/s [22,27,29]. The diffusion constant D_Cafree_ = 270 μm^2^/s being used in the model is in the range.

Using FRAP (fluorescence recovery after bleach) of intra-SR Ca^2+^ indicator Fluo-5N, Wu and Bers estimated the diffusion of Ca^2+^ in the SR of rabbit cardiac myocytes to be about 60 μm^2^/s [72]. However, another study estimated a much smaller value, 8–9 μm^2^/s in rat and guinea pig myocytes [73]. Swietach and co-workers suggested that the slow diffusion for Ca^2+^ in the SR helps to explain the long recovery time for [Ca^2+^]_jSR_ within 100–200 ms. In addition, the authors suggested that the high value measured by Wu and Bers did not consider the SR Ca^2+^ leak during measurement. A recent study by Picht and co-workers supported the previous result in Bers’ group and rejected that claim [74]. A study by Sobie and Lederer supported the results of Bers’ group in that their simulation data can reproduce simulation results with D_Casr_ = 60 μm^2^/s, rather than with D_Casr_ = 20 μm^2^/s [75]. In order to explain the slow recovery during Ca^2+^ sparks, e.g., 161 ms by [76], given the fast diffusion of Ca^2+^ in the SR, one possible hypothesis is that the duration of SR Ca^2+^ release, time-to-blink nadir, is longer than time-to-spark peak [75,77]. The authors suggested some irregularities in the structure of the RyR2 cluster, e.g., involving rogue RyR2s.

Another factor for the long recovery time is not only the sharp depletion of [Ca^2+^] in the jSR compared to the network SR, but also the partial depletion of [Ca^2+^] in the neighboring nSR grid points. The released Ca^2+^ includes not only the free calcium but also the calcium that binds to CSQ. The amount of Ca^2+^ that binds to CSQ in the cardiac muscle was estimated to be about 50% [52]; although this value is likely to vary with species and experimental conditions. Terentyev and co-workers applied an acute, 3-fold reduction in CSQ2 in quiescent rats and found about a 2-fold decrease in SR Ca^2+^; without change in free intra-SR [Ca^2+^], which suggested 70% of Ca^2+^ was bound to CSQ2 [78,79]. Given the depletion of [Ca^2+^]_jSR_ from 1000 μM to ~100 μM during a Ca^2+^ spark, as suggested by a previous version of our current model [20] and a recent study [19], and the binding constant between CSQ and Ca^2+^ is about 400 μM, a significant amount of Ca^2+^ unbound from CSQ are released. In addition, we also observe a significant Ca^2+^ reduction in the neighboring nSR (~500 μM). Thus, the recovery time for [Ca^2+^]_jSR_ should be long enough to provide an adequate amount of Ca^2+^ to refill the free [Ca^2+^]_jSR_, to neighboring nSR, including Ca^2+^ to bind to [CSQ]. This, not including the possible rate-limiting effect between the jSR and nSR due to geometrical structure, can be used to explain the long recovery time, given the diffusion constant of [Ca^2+^]_SR_ at 60 μm^2^/s. Thus, in the model, we chose D_Casr_ = 60 μm^2^/s.

The diffusion of Fluo-3 constant 90 μm^2^/s was used in the model. This is in agreement with the measurement [80] that is almost 5-fold larger than what has been measured earlier in skeletal muscle [81]. The role of ATP mobility and its serving as a Ca^2+^ buffer has not been considered in the model [82]. Based on molecular weight, calmodulin (Calm) is expected to diffuse with about one order lower than that of calcium. Even though other studies have modeled it as a mobile buffer with a small diffusion constant: 20–40 μm^2^/s, calmodulin is modeled as a stationary buffer in our model.

### 2.7. Model Formulation in the Spatial Cell

**Figure 3:**
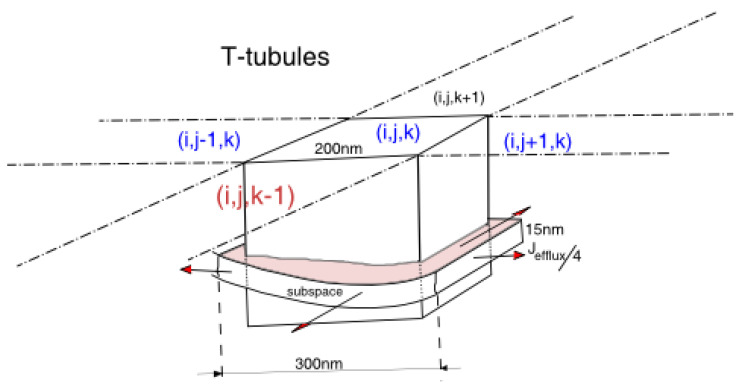
The schematic diagram shows a grid point as a cube of size 200 nm on each dimension in the 3-dimensional space. In this cube, we put a single dyad. Assuming that the width of the dyad is 300 nm, we model the efflux of calcium flowing into 4 adjacent grid points, and it is equally split into 4 parts. The grid location is given by the coordinate (i,j,k).

At each grid point, the model contains the concentrations of the chemical species in the myoplasm, and in the network SR. Each grid point contains a myoplasmic fraction and SR fraction (Figure 3). In the myoplasmic volume part of a grid point, the chemical species are calcium, calcium bound to fluorescent indicator dye and other calcium-bound buffers. Depending upon the location of the grid point, i.e., if it resides at the external SL or the T-tubule, there can be other species, e.g., SL buffer. Due to the fast diffusion rate of the membrane potential V_m_, it is assumed that the transmembrane potential V_m_ is spatially uniform distributed across the cell. The dynamics of transmembrane potential is derived from the ionic currents in the form:

(5) $$\frac{{\mathrm{dV}}_{m}}{\mathrm{dt}}=-\frac{1}{C_{\mathrm{sc}}}\left(\underset{i=\mathrm{ion}}{\sum }I_{i}\right)+I_{\mathrm{app}}$$

where $I_{\mathrm{app}}$ is the stimulus current. At this scope of the study, it is assumed that there is no ionic exchange between the extracellular media and the inside of the cell. However, for completeness of the model description, all the mathematical equations are described.

(6) $$\underset{i=\mathrm{ion}}{\sum }I_{i}=I_{\mathrm{Na}}+\frac{I_{\mathrm{dhpr}}^{T}}{A_{m}}+I_{K1}+I_{\mathrm{Kss}}+I_{\mathrm{Ktof}}+I_{\mathrm{Ktos}}+\bar{I_{\mathrm{NCX}}}+I_{\mathrm{Na}/K}+\bar{I_{\mathrm{pmca}}}+I_{\mathrm{bNa}}+\bar{I_{\mathrm{bCa}}}+I_{\mathrm{bK}}$$

where

(7) $$I_{\mathrm{dhpr}}^{T}=\underset{i=\mathrm{indexCRU}}{\sum }I_{\mathrm{dhpr}}^{\left(i\right)}$$

(8) $$\bar{I_{\mathrm{NCX}}}=\frac{\sum_{i=1}^{\#\mathrm{grids}-\mathrm{as}-\mathrm{membrane}}I_{\mathrm{NCX}}^{\left(i\right)}}{\#\mathrm{grids}-\mathrm{as}-\mathrm{membrane}}$$

(9) $$\bar{I_{\mathrm{bCa}}}=\frac{\sum_{i=1}^{\#\mathrm{grids}-\mathrm{as}-\mathrm{membrane}}I_{\mathrm{bCa}}^{\left(i\right)}}{\#\mathrm{grids}-\mathrm{as}-\mathrm{membrane}}$$

(10) $$\bar{I_{\mathrm{pmca}}}=\frac{\sum_{i=1}^{\#\mathrm{grids}-\mathrm{as}-\mathrm{membrane}}I_{\mathrm{pmca}}^{\left(i\right)}}{\#\mathrm{grids}-\mathrm{as}-\mathrm{membrane}}$$

In the current model, only the spatial distribution of calcium and calcium-bound species are considered. In the myoplasm, calcium is buffered by the endogenous buffers (calmodulin (Calm), troponin-C (Trpn), SL, SR membrane buffers) or exogeneous buffers (Fluo-3 (F)) with the kinetics based on [83]:

(11) $$J_{\mathrm{buffer}}=J_{\mathrm{CaTrpn}}+J_{\mathrm{CaCM}}+J_{\mathrm{CaSR}}+J_{\mathrm{CaF}}+J_{\mathrm{CaSL}}$$

with CaTrpn = calcium-bound to troponin, CaCalm = calcium-bound calmodulin, CaSR = calcium-bound to SR buffer, CaF = calcium-bound to Fluo-3. Of these, calmodulin, the SL, SR membrane buffers and troponin are assumed to be stationary and are described by the following ordinary differential equations:

(12) $$\frac{d\left[\mathrm{CaCalm}\right]}{\mathrm{dt}}=J_{\mathrm{Calm}}=k_{\mathrm{Calm}}^{+}\left({\left[\mathrm{Ca}\right]}_{\mathrm{myo}}\right)\left({\left[\mathrm{Calm}\right]}_{T}-{\left[\mathrm{CaCalm}\right]}_{\mathrm{myo}}\right)-k_{\mathrm{Calm}}^{-}{\left[\mathrm{CaCalm}\right]}_{\mathrm{myo}}$$

(13) $$\frac{d\left[\mathrm{CaSR}\right]}{\mathrm{dt}}=J_{\mathrm{CaSR}}=k_{\mathrm{SR}}^{+}\left({\left[\mathrm{Ca}\right]}_{\mathrm{myo}}\right)\left({\left[\mathrm{SR}\right]}_{T}-{\left[\mathrm{CaSR}\right]}_{\mathrm{myo}}\right)-k_{\mathrm{SR}}^{-}{\left[\mathrm{CaSR}\right]}_{\mathrm{myo}}$$

(14) $$\frac{d\left[\mathrm{CaSL}\right]}{\mathrm{dt}}=J_{\mathrm{CaSL}}=k_{\mathrm{SL}}^{+}\left({\left[\mathrm{Ca}\right]}_{\mathrm{myo}}\right)\left({\left[\mathrm{SL}\right]}_{T}-{\left[\mathrm{CaSL}\right]}_{\mathrm{myo}}\right)-k_{\mathrm{SL}}^{-}{\left[\mathrm{CaSL}\right]}_{\mathrm{myo}}$$

(15) $$\frac{d\left[\mathrm{CaTrpn}\right]}{\mathrm{dt}}=J_{\mathrm{Trpn}}=k_{\mathrm{Trpn}}^{+}\left({\left[\mathrm{Ca}\right]}_{\mathrm{myo}}\right)\left({\left[\mathrm{Trpn}\right]}_{T}-{\left[\mathrm{CaTrpn}\right]}_{\mathrm{myo}}\right)-k_{\mathrm{Trpn}}^{-}{\left[\mathrm{CaTrpn}\right]}_{\mathrm{myo}}$$

The exogenous buffer Fluo-3 are mobile buffers and are describe by the following differential equations:

(16) $$\frac{\partial {\left[\mathrm{CaF}\right]}_{\mathrm{myo}}}{\partial t}=J_{\mathrm{CaF}}+\nabla^{2}{\left[\mathrm{CaF}\right]}_{\mathrm{myo}}$$

(17) $$\frac{\partial {\left[F\right]}_{\mathrm{myo}}}{\partial t}=-J_{\mathrm{CaF}}+\nabla^{2}{\left[F\right]}_{\mathrm{myo}}$$

where the fluxes are:

(18) $$J_{\mathrm{CaF}}=k_{F}^{+}\left({\left[\mathrm{Ca}\right]}_{\mathrm{myo}}\right)\left[F\right]-k_{F}^{-}\left[\mathrm{CaF}\right]$$

Free calcium and mobile buffers passively diffuse from one grid point to the neighboring ones following Fickian diffusion laws. Combined with the above fluxes, the following partial differential equations describe these model variables:

(19) $$\frac{\partial {\left[{\mathrm{Ca}}^{2+}\right]}_{\mathrm{myo}}}{\partial t}=J_{\mathrm{ryr}}-J_{\mathrm{buffer}}-J_{\mathrm{serca}}+\nabla^{2}{\left[{\mathrm{Ca}}^{2+}\right]}_{\mathrm{myo}}$$

(20) $$\frac{\partial {\left[{\mathrm{Ca}}^{2+}\right]}_{\mathrm{nsr}}}{\partial t}=J_{\mathrm{serca}}+J_{\mathrm{refill}}+\nabla^{2}{\left[{\mathrm{Ca}}^{2+}\right]}_{\mathrm{nsr}}\;$$

The calcium in the jSR is replenished from the nSR by a diffusive flux ($J_{\mathrm{refill}}$) which has been described by [84]. Even though Ca^2+^ release via RyR2 from the jSR can spread by more than one grid point, the jSR is treated as a single volume. Thus, the changes in [Ca^2+^]_jSR_ at the i^th^ release site is described by the following ordinary differential equation:

(21) $$\frac{\partial {\left[{\mathrm{Ca}}^{2+}\right]}_{\mathrm{jsr}}^{i}}{\partial t}=\beta_{\mathrm{jsr}}\left(J_{\mathrm{refill}}-J_{\mathrm{RyR}2}\right)/\lambda_{\mathrm{jsr}}$$

where fast buffering is assumed in the jSR, and λ_jsr_ = V_jsr_/V_myo_ is the volume fraction.

(22) $$\beta_{\mathrm{jsr}}=1/(1+\left(B_{\mathrm{jsr}}^{T}\times {\mathrm{Km}}_{\mathrm{jsr}}\right)/(\left({\mathrm{Km}}_{\mathrm{jsr}}+\left[{\mathrm{Ca}}^{2+}{]}_{\mathrm{jsr}}^{i}{)}^{2}\right)\right)$$

### 2.8. Computational Methods

The model is fully stochastic in terms of the channel gating of RyR2s and LCCs. The program was mainly written in Fortran and CUDA Fortran, using the CUDA programming toolkit to run on Nvidia Fermi GPU, with some parts written in C++. The Euler method was used to solve the partial-differential equations (PDEs) of diffusion-reaction, and other ordinary differential equations (ODEs). The adaptive time-step ranging from 10 ns to 1 µs was used. When there is some activity, due to channel gating, the time-step will be reduced for numerical stability. The units in the systems are as follows: transmembrane potential—mV, membrane currents—µA/cm^2^, the ionic concentration—µM (defined based on the corresponding volume), time-second (s). In the spatial model, the forward difference in time and central difference in space derivatives yields:

(23) $$\begin{array}{cc} \frac{u_{\mathrm{i,j,k}}^{t+\Delta t}-u_{\mathrm{i,j,k}}^{t}}{\Delta t}= & f(u_{\mathrm{i,j,k}}^{t},t)+D_{x}\left(\frac{u_{i+1,j,k}^{t}-2u_{\mathrm{i,j,k}}^{t}+u_{i-1,\mathrm{j,k}}^{t}}{{\left(\Delta x\right)}^{2}}\right)+D_{y}\left(\frac{u_{i,j+1,k}^{t}-2u_{\mathrm{i,j,k}}^{t}+u_{i,j-1,k}^{t}}{{\left(\Delta y\right)}^{2}}\right)\\ & \;+D_{z}\left(\frac{u_{i,j,k+1}^{t}-2u_{\mathrm{i,j,k}}^{t}+u_{i,j,k-1}^{t}}{{\left(\Delta z\right)}^{2}}\right)\frac{u_{\mathrm{i,j,k}}^{t+\Delta t}-u_{\mathrm{i,j,k}}^{t}}{\Delta t}\\ & \;=f(u_{\mathrm{i,j,k}}^{t},t)+D_{x}\left(\frac{u_{i+1,\mathrm{j,k}}^{t}-2u_{\mathrm{i,j,k}}^{t}+u_{i-1,\mathrm{j,k}}^{t}}{{\left(\Delta x\right)}^{2}}\right)+D_{y}\left(\frac{u_{i,j+1,k}^{t}-2u_{\mathrm{i,j,k}}^{t}+u_{i,j-1,k}^{t}}{{\left(\Delta y\right)}^{2}}\right)\\ & \;+D_{z}\left(\frac{u_{i,j,k+1}^{t}-2u_{\mathrm{i,j,k}}^{t}+u_{i,j,k-1}^{t}}{{\left(\Delta z\right)}^{2}}\right) \end{array}$$

(24) $$\begin{array}{c} \frac{u_{\mathrm{ijk}}^{p+1}-u_{\mathrm{ijk}}^{p}}{\Delta t}=f(u^{p},t)\\ \;\;\;\;\;\;+D(\frac{u_{i+1,j,k}^{p}-2u_{\mathrm{ijk}}^{p}+u_{i-1,j,k}^{p}}{{\left(\Delta x\right)}^{2}}+\frac{u_{i,j+1,k}^{p}-2u_{\mathrm{ijk}}^{p}+u_{i,j-1,k}^{p}}{{\left(\Delta y\right)}^{2}}+\frac{u_{i,j,k+1}^{p}-2u_{\mathrm{ijk}}^{p}+u_{i,j,k-1}^{t}}{{\left(\Delta z\right)}^{2}})\frac{u_{\mathrm{ijk}}^{p+1}-u_{\mathrm{ijk}}^{p}}{\Delta t}\\ \;\;\;\;\;\;=f(u^{p},t)+D\left(\frac{u_{i+1,j,k}^{p}-2u_{\mathrm{ijk}}^{p}+u_{i-1,j,k}^{p}}{{\left(\Delta x\right)}^{2}}+\frac{u_{i,j+1,k}^{p}-2u_{\mathrm{ijk}}^{p}+u_{i,j-1,k}^{p}}{{\left(\Delta y\right)}^{2}}+\frac{u_{i,j,k+1}^{p}-2u_{\mathrm{ijk}}^{p}+u_{i,j,k-1}^{t}}{{\left(\Delta z\right)}^{2}}\right) \end{array}$$

where u represents the species concentration in a single grid point.

(25) $$\begin{array}{c} u_{\mathrm{ijk}}^{p+1}=u_{\mathrm{ijk}}^{p}+\Delta t\{f(u,t)\\ \;\;\;\;\;\;\;+D(\frac{u_{i+1,j,k}^{p}-2u_{\mathrm{ijk}}^{p}+u_{i-1,j,k}^{p}}{{\left(\Delta x\right)}^{2}}+\frac{u_{i,j+1,k}^{p}-2u_{\mathrm{ijk}}^{p}+u_{i,j-1,k}^{p}}{{\left(\Delta y\right)}^{2}}+\frac{u_{i,j,k+1}^{p}-2u_{\mathrm{ijk}}^{p}+u_{i,j,k-1}^{t}}{{\left(\Delta z\right)}^{2}})\}u_{\mathrm{ijk}}^{p+1}\\ \;\;\;\;\;\;\;=u_{\mathrm{ijk}}^{p}\\ \;\;\;\;\;\;\;+\Delta t\{f(u,t)\\ \;\;\;\;\;\;\;+D\left(\frac{u_{i+1,j,k}^{p}-2u_{\mathrm{ijk}}^{p}+u_{i-1,j,k}^{p}}{{\left(\Delta x\right)}^{2}}+\frac{u_{i,j+1,k}^{p}-2u_{\mathrm{ijk}}^{p}+u_{i,j-1,k}^{p}}{{\left(\Delta y\right)}^{2}}+\frac{u_{i,j,k+1}^{p}-2u_{\mathrm{ijk}}^{p}+u_{i,j,k-1}^{t}}{{\left(\Delta z\right)}^{2}})\right\} \end{array}$$

For a single cell, the Neumann boundary condition is used, i.e., no flux:

(26) $$u_{0,j,k}^{\;p}=u_{1,j,k}^{p}\;\mathrm{and}\;u_{X+1,j,k}^{\;p}=u_{X,j,k}^{p}$$

(27) $$u_{i,0,k}^{\;p}=u_{i,1,k}^{p}\;\mathrm{and}\;u_{i,Y+1,k}^{\;p}=u_{i,Y,k}^{p}$$

(28) $$u_{i,j,0}^{\;p}=u_{1,j,1}^{p}\;\mathrm{and}\;u_{i,j,Z+1}^{\;p}=u_{X,j,Z}^{p}$$

To handle the extremely large data generated by the 3D model, the HDF5 (hierarchical data format) library was used [85], with the data exported in a compressed format that can be extracted later for further data analysis. The IDL language (data visualization software, 2014) was used to perform all data visualization and data analysis. In this study of calcium waves, the two ions (K^+^, Na^+^) were kept constant for the duration of the simulation. Similarly, the corresponding currents were also modeled as uniform [63].

In the 3D model, several 3D data arrays need to be created, e.g., data for cytosolic calcium, myoplasmic calcium, calcium-bound to fluorescence, free fluorescence. However, there are certain data that are not ‘everywhere’ but are only present at a certain number of grid points. Many of them are species that reside on the sarcolemma membranes, e.g., SL buffer, ionic channels (K^+^, Na^+^, LCC) and pumps/exchangers (NCX, PMCA, Na^+^/K^+^). To save memory and to enhance the performance, a single 3D data array called grid point data is used. Each element of this array is a 32-bit value, where we split them into groups of 4 bits. Each group of bits tells us some information about that grid point.

Bit 0–3: index to the array that tells NCX distribution
Bit 4–7: index to the array that tells SERCA distribution
Bit 8–11: index to the array that tells SR buffer distribution
Bit 12–15: index to the array that tells Troponin-C distribution
Bit 16–19: index to the array that tells the grid-type (MEMBRANE, INNER-GRIDPOINT, OUTER-GRIDPOINT, NUCLEUS, MITO). Currently, we only use MEMBRANE (both SL and T-tubule) and INNER-GRIDPOINT and OUTER-GRIDPOINT (stencil grid point).
Bit 20–31: reserved (for future use)

This significantly reduces the memory usage. From six 3D arrays, we now only need the same number of 1D arrays of a few data elements, and a single 3D array. In the current study, only channels, buffers related to Ca^2+^ are modeled spatially. The arrays that describe the SL buffer distribution, SR buffer distribution, and NCX distribution contain just one (in the case of uniform distribution), or two (in the case we have high and low distribution) data elements. The value in each array represents the concentration of the corresponding species in each grid point. This is important due to the limited memory on CUDA-capable GPUs, and to reduce the overhead of memory access, where reading data from global memory is very expensive compared to reading data on constant memory of much smaller sizes as bit-operations are very fast.

## 3. Results

### 3.1. Ca^2+^ Transient

**Figure 4:**
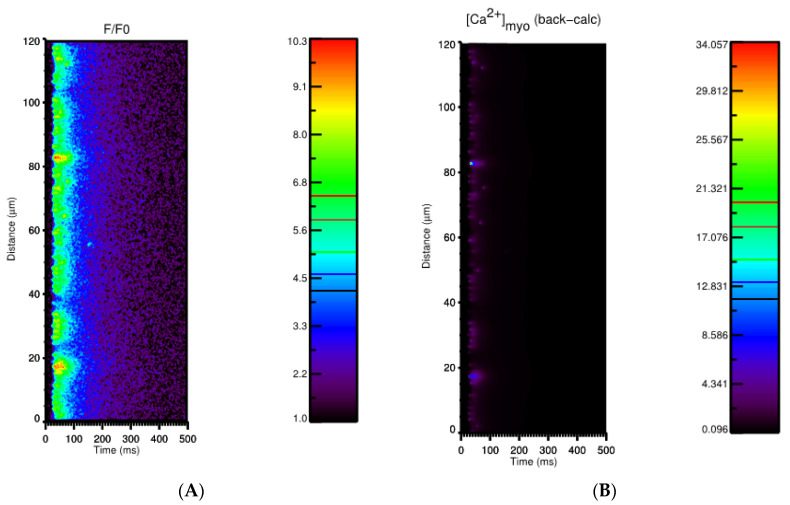

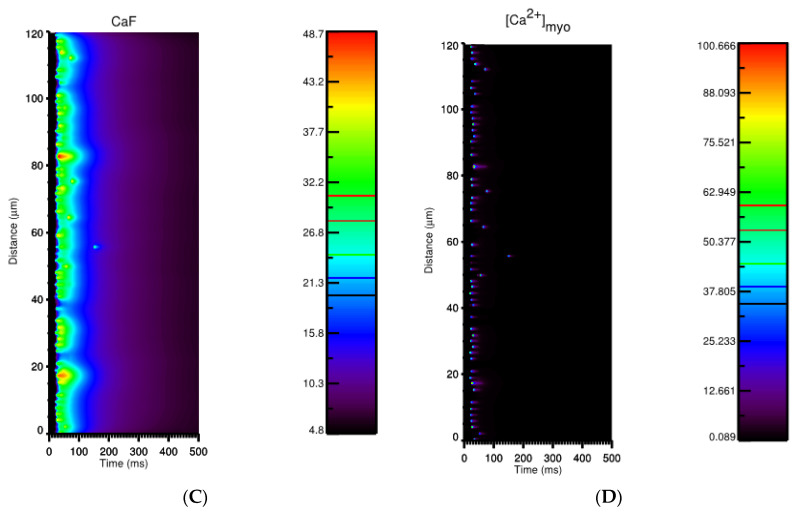
Using the simulation data, a multitude of information can be extracted. Apart from a snapshot of a pseudo-line scan calcium transient during Ca^2+^ transient along the longitudinal direction, as shown in (**A**), we are able to show (**B**) the dynamics of free calcium content, (**C**) the dynamics of calcium-bound fluorescent, (**D**) the dynamics of total calcium content.

A line-scan image along the longitudinal direction during the Ca^2+^ transient is shown in Figure 4. The current model allows the investigation of the level of Ca^2+^ directly without using Ca^2+^-bound fluorescent intensities. Even though the value F/F0 is in agreement with experimental data, compared to the result using the back-calculation formula given by [1], the peak of free Ca^2+^ is about 3 times higher than the value estimated. This means that the calculated calcium from the experiment underestimated the amount of level free calcium in the cell. This emphasizes that the functional role of local Ca^2+^ near the Ca^2+^ release site (at the z-line) in the cell due to the restricted space can regulate different cellular signals. Aside from a snapshot of a pseudo-line scan calcium transient during a Ca^2+^ transient along the longitudinal direction (A), we also demonstrate the dynamics of free calcium content in (B), the dynamics of calcium-bound fluorescence in (C), and the dynamics of total calcium content in (D).

### 3.2. Ca^2+^ Spark Induced Ca^2+^ Sparks

The current model has the following key features: (1) The gating of ion channels is modeled stochastically with the RyR2 kinetics fitted to give the proper fidelity of spark generation that matches the experimental data, i.e., ~100 sparks/cell/s at rest. (2) The non-uniform distribution of SL, SR buffers and NCX were incorporated in the model to reflect experimental reality. (3) The dynamics of calcium buffering in the subspace is modeled explicitly, without using fast binding assumption. (4) The ultra-fast MCMC method allows us to obtain a better statistic of spark-induced spark compared to other studies which only enable the examination one, two, or a few pairs of CRUs at a time. The first simulation series explores the role of different factors, e.g., [Ca^2+^]_myo_, [Ca^2+^]_SR_, and CRU distances in spark triggering. The result was collected based on 1416 simulation cases.

**Figure 5:**
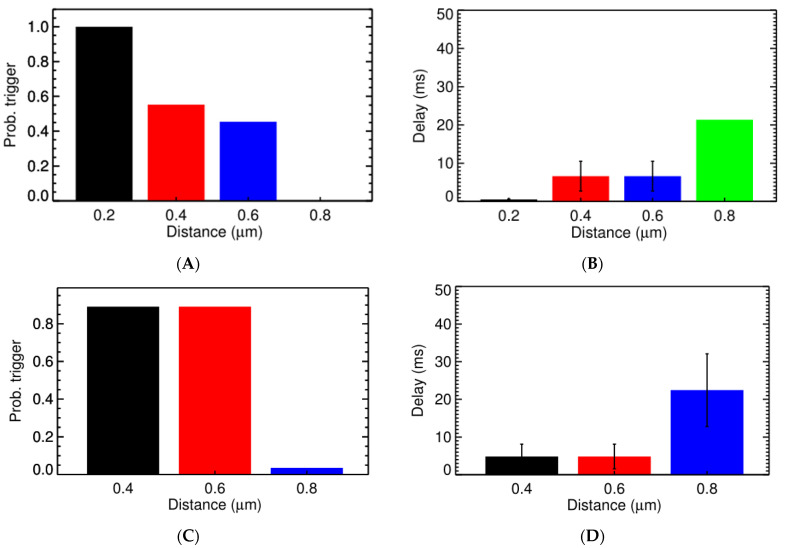
The probability of one CRU triggering the neighboring one at different distance and the delay. (**A**) The probability of triggering a Ca^2+^ spark under normal diastolic conditions ([Ca^2+^]_myo_ = 0.096 µM, and [Ca^2+^]_nsr_ = 1.02 mM). (**B**) The Ca^2+^ spark triggering delay under normal conditions. (**C**) The probability of triggering a Ca^2+^ spark under high cytosolic calcium ([Ca^2+^]_myo_ = 0.4 µM, and [Ca^2+^]_nsr_ = 1.02 mM). (**D**) The Ca^2+^ spark triggering delay under high cytosolic calcium.

**Figure 6:**
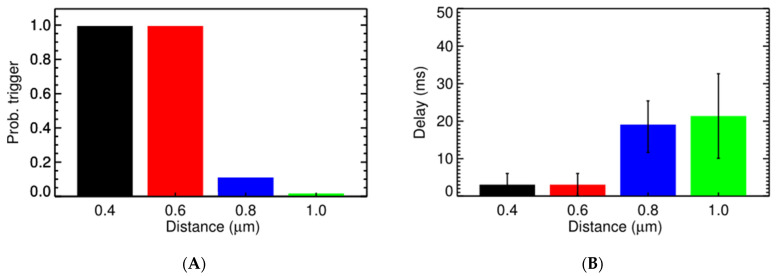

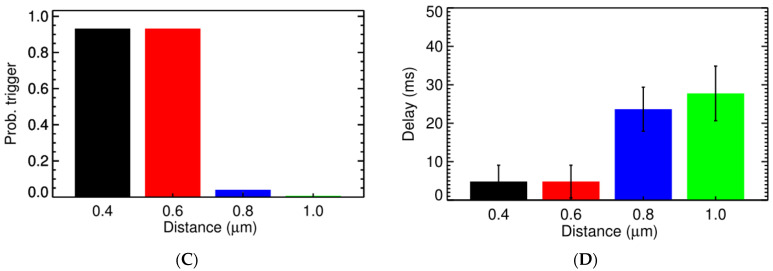
The probability of one CRU triggering the neighboring one at different distances and the delay. (**A**) The probability of triggering a spark at the second CRU at different distances from an activated CRU under the high overload condition ([Ca^2+^] = 0.156 µM, and [Ca^2+^]_nsr_ = 1.70 mM). (**B**) The delay in triggering the second CRU under that high overload condition. (**C**) The probability of triggering a spark at the second CRU at different distances from an activated CRU under the low overload condition ([Ca^2+^]_myo_ = 0.156 µM, and [Ca^2+^]_nsr_ = 1.30 mM). (**D**) The delay in triggering the second CRU under that low overload condition.

Under normal conditions, a sharp transition occurs when the CRU distance goes beyond 0.6 µm (Figure 5) with the probability for triggering activation of a CRU (P_o,trigger_) at 0.8 µm being only 0.14% and the delay in time being 21 ms on average, as shown in Figure 5A,B. This is in agreement with experimental data where Ca^2+^ sparks are the local release of calcium that span a restricted space, without affecting the neighboring sites [1]. The increase of cytosolic calcium, to 0.1564 µM, brings the P_o,trigger_ for the CRU at distance 0.6 µm from 45.40% to 58.41%. However, at [Ca^2+^]_myo_ = 0.4 µM, it significantly increases the P_o,trigger_ which is now 89.12% for CRU at 0.6 µm apart and is 3.56% for the CRU at 0.8 µm apart. Along with this, we see a reduction in the delay time, which reduces from 6.6 ms for the CRU at 0.6 µm apart in Figure 5A,B, to 4.8 ms for the CRU at 0.6 µm apart in Figure 5C,D. A similar result can be achieved when increasing [Ca^2+^]_sr_ (Figure 6). In particular, we tested under two overload conditions with different levels of [Ca^2+^]_nsr_. In Figure 6A,B, the high overload conditions are shown ([Ca^2+^]_myo_ = 0.156 µM, and [Ca^2+^]_nsr_ = 1.70 mM), and in Figure 6C,D, the low overload condition is shown ([Ca^2+^]_myo_ = 0.156 µM, and [Ca^2+^]_nsr_ = 1.30 mM). In each condition, we measure the probabilities of one CRU triggering the neighboring ones at different distances and the delay upon which condition it occurs under. The main cluster can trigger the neighboring cluster at 0.6 µm apart with high fidelity (almost 99%) and very small delay (<8 ms). This strongly suggests the role of small clusters in bridging the gap between CRUs in generating calcium waves across all tested conditions.

To induce higher SR calcium, ref. [86] applied anti-phospholamban (APL) antibodies. The results showed that calcium sparks increase from ~4 events/100 µm to ~7 events/100 µm and remains at this level during the experiment. Using [Ca^2+^]_o_ = 10 mM, a 4-fold increase in Ca^2+^ spark was observed [2]. In the cardiac cell, Ca^2+^ binds to calsequestrin (CSQ) to directly regulate RyR2 gating from the lumenal side. Even though CSQ can exist in multiple forms (monomers, dimers and multimers), the monomer undertakes the regulatory function in RyR2 [87]; while the role of multimers in RyR2 regulation has not been confirmed other than serving as Ca^2+^ buffers [88].

During Ca^2+^ overload, a significant amount of SR Ca^2+^ is bound to CSQ in oligomer forms. In the current model, lumenal dependency is modeled as a function of [Ca^2+^]_jSR_, not [Ca^2+^/CSQ]. The multiple forms of CSQ, i.e., dimers and multimers, which can function as Ca^2+^ buffers to hold more SR Ca^2+^ during Ca^2+^ overload have not been modeled. With a fixed amount of CSQ at the jSR, we suggest that when [Ca^2+^]_jSR_ exceeds a certain amount, the dependency on [Ca^2+^]_jSR_ saturates. Thus, the lumenal function is revised to match the spark frequency from the experimental results at the Ca^2+^ overload condition, while also allowing a higher [Ca^2+^]_SR_ that can trigger a Ca^2+^ wave. The lumenal function is changed from:

$$\Theta =k_{0}\times {\left[{\mathrm{Ca}}^{2+}\right]}_{\mathrm{jSR}}+k_{1}$$

to:

$$\Theta =k_{0}\times \min\left({\mathrm{Ca}}_{\max,}{\left[{\mathrm{Ca}}^{2+}\right]}_{\mathrm{jSR}}\right)+k_{1}$$

with Ca_max_ = 1.13 mM being the saturation value of [Ca^2+^]j_SR_ that was selectedwas to be in agreement with the previous experiment.

The difference in P_o,trigger_ between the two cases is small, as shown in Table 1 and Table A1 in Appendix A, suggesting that the P_o,trigger_ is strongly influenced by the Ca^2+^ diffusing from the neighboring release site. Based on the measurement in Figure 4C of [59], a significant number of transversal CRUs have nearest neighbors that fall into the 0.7 µm range (21%), which may allow multiple CRUs to be activated on the same z-disc.

**Figure 7:**
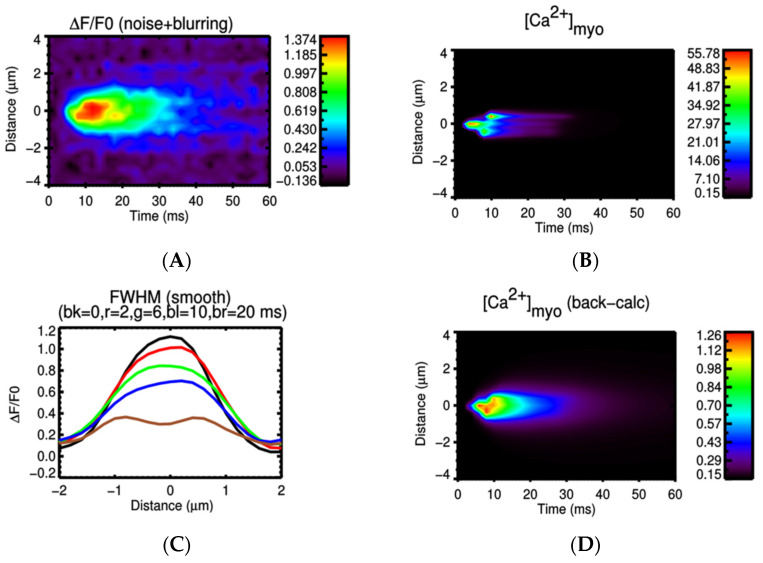
(**A**) A simulated calcium spark. (**B**) Free calcium shows the underlying structure of the release site (the delayed activation of the two satellite clusters are invisible under fluorescence profile. (**C**) The profile of a calcium spark giving FWHM = 1.85 um (each color represents the snapshot at different time points after the peak (e.g., bk = 0 means the black line at 0 ms is delayed). (**D**) The free calcium profile using back-calculation method agrees with experimental estimates, however, it underestimates the real free myoplasmic calcium amplitude.

**Figure 8:**
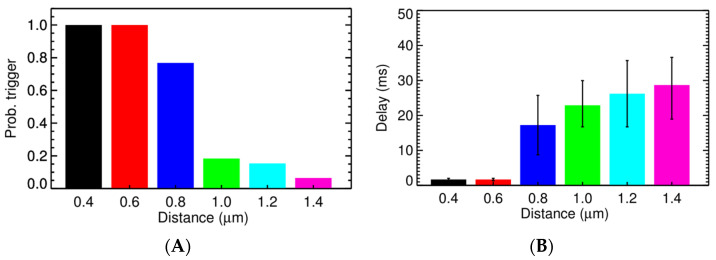
The probability of one CRU triggering the neighboring CRU at (**A**) different distance and (**B**) the delay. The overload conditions ([Ca^2+^]_myo_ = 0.156 µM, and [Ca^2+^]_nsr_ = 1.30 mM) where each CRU has 3 satellite clusters of 10 RyR2s, each at distance 0.2 µm were used.

Experimental studies using high resolution imaging showed that RyR2s are organized in subclusters that can function as single functional CRUs [77,89,90,91]. In this study, by using a big cluster with 36 RyR2 and two smaller satellite clusters of 15 RyR2 at 0.150 µm apart, the realistic FWHM = 1.85 μm was simulated. In Figure 7A, there is a pseudo-line scan image showing a single calcium spark, this is the result of applying Gaussian noise and incorporating the effect of optical blurring to the calculated $\Delta F/F_{0}\;$which is a dynamic variable in the model. The FWHM of the calcium spark is calculated after applying smoothing (3 × 3 window) filtering. Using the computational model, it enables us to record the underlying calcium dynamics which shows the contributions from multiple subclusters. This is, however, invisible if back-calculating the value from the recorded fluorescent signal occurs, as is done in the experimental protocol. This model, for the first time, shed light onto the unresolved question of the FWHM that has not been replicated in previous models. The role of these satellite clusters in spark-induced spark triggering is tested by modeling each CRU with 3 satellite clusters of size 10 RyR2s at 0.2 µm apart. Figure 8 shows that the satellite clusters help boost the chance for spark-induced sparks. Compared to the result in Figure 6B, the CRU can now trigger a neighboring CRU at a further distance, with a higher probability for the ones at the same distances as used in the earlier case. This is in agreement with the study by the authors of [92], in which they suggested that blocking small, non-spark producing clusters of RyR2 using ruthenium red (RuR) are important to the process of Ca^2+^ wave propagation.

### 3.3. Ca^2+^ Spark Induced Ca^2+^ Waves

In the study by Cheng and co-workers [2], increasing extracellular from 1 mM to 10 mM elicited a 4-fold increase in Ca^2+^ sparks, with spark amplitude and spark size increasing 4.1-fold and 1.7-fold accordingly. These macro Ca^2+^ sparks also served as the sites of wave initiation (65%), indicating that the macrosparks may represent aborted waves involving propagation between release sites. Our study, which is in agreement with these studies, also suggests that the Ca^2+^ spark observed is the result of calcium release, not from a single CRU but multiple CRUs, and thus the mass of Ca^2+^ release from multiple CRUs is considered strong enough to trigger the wave in most cases.

The general mechanism for Ca^2+^ wave propagation is the fire-diffuse-fire model [24,25] with an estimated wave velocity of 67 µm/s with CRUs at d = 2.0 µm apart. For sustained Ca^2+^ wave propagation, there are two important factors: first the diffusing Ca^2+^ from one Z-line should be able to activate the CRU in the next Z-line, and then calcium release from this CRU should be able to activate the neighboring ones in the same Z-line, and the process repeats. Given the shorter nearest neighbor distances for CRU on the same Z-line, in Figure 4, the CRUs on the same Z-line should be activated first during a Ca^2+^ wave initiation. This result is in agreement with the prediction that a single spark is unlikely to trigger a spark at the next Z-line [26,93].

The previous section already provided the P_o,trigger_ at which a Ca^2+^-spark from one CRU can induce the activation of the neighboring CRU in the same Z-line under the different conditions of [Ca^2+^]_myo_, [Ca^2+^]_nsr_, different distances and of the presence of satellite RyR2. The second factor contributing wave formation along the longitudinal direction at which the distance between the CRUs is much longer at 1.4–2.2 µm. The question is under what condition can this happen? This is determined by two factors: the number of CRUs activated on one Z-line and the mass of Ca^2+^ release, which depends critically on the level of Ca^2+^ overload.

**Figure 9:**
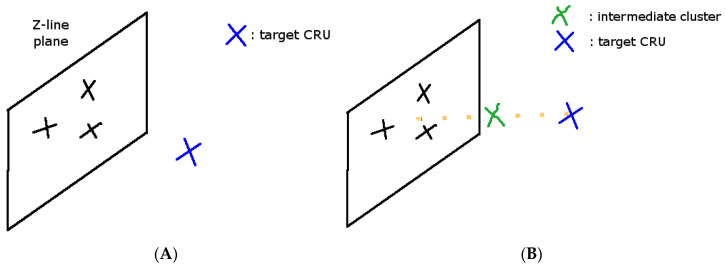
**(A)** Simulation geometry to study spark-induced waves (X = CRU location). Black X = the activated CRU, Blue X = the CRU to be activated by diffusing Ca^2+^. (**B**) The proposed simulation geometry with an intermediate cluster (Green X = intermediate RyR2 cluster).

A simulation study placing a few CRUs on the same Z-line that are activated, and another CRU at the other end of the sarcomere, was performed. Instead of modeling the activation of a single CRU, in this case, a model at which a few ‘black’ CRUs are activated, allowed the analysis of the P_o,trigger_ of the ‘blue’ CRU (Figure 9).

Simulations with [Ca^2+^]_SR_ = 1.3 mM with 9 activated CRUs failed to produce enough Ca^2+^ release to activated nearby release sites. Given the spatial resolution of the confocal microscope is 10 times larger than the size of a junctional SR (0.06 fL vs. 0.008 fL), it is practically impossible to accurately detect the level of Ca^2+^ in the jSR. In addition, fluo-5N, the dye being used to estimate [Ca^2+^]_jSR_, has the Ca^2+^ affinity ~400 μM, which is 2–3 times smaller than the diastolic level of [Ca^2+^]_SR_. Thus, estimating the level of [Ca^2+^]_SR_ overload is currently impossible [27]. To estimate the total [Ca^2+^]_SR_ during a Ca^2+^ overload, it requires detailed modeling of CSQ in multiple forms which is not available in the current model. Therefore, we focus on the number of activated sites. Nevertheless, we were able to see a full and repetitive Ca^2+^ wave with [Ca^2+^]_SR_ = 1.7 mM and [Ca^2+^]_myo_ = 0.156 μM. This seems to provide a “safety factor” where normal [Ca^2+^]_SR_ cannot produce Ca^2+^ activation of adjacent sites to form waves.

Izu and co-workers used a 5–7 supercluster [26]. Each super-CRU releases 10–20 pA, compared to the typical 3 pA current [1,94]. This maps to the number of 15–27 CRUs being fired at the same time to achieve the wave velocity of 126 μm/s. In our model, 7–9 CRUs were enough to trigger a Ca^2+^ wave. Thus, the spark model in our system, the triggering of a Ca^2+^ wave during Ca^2+^ overload is more likely to happen. In addition, Izu et al. (2001) used a two-dimensional space which clearly enhances the Ca^2+^ wave propagation compared to 3D. The general condition to test is [Ca^2+^]_myo_ = 0.156 µM, [Ca^2+^]_sr_ = 1.7 mM, and the statistics were collected based on 144 trial cases.

**Figure 10:**
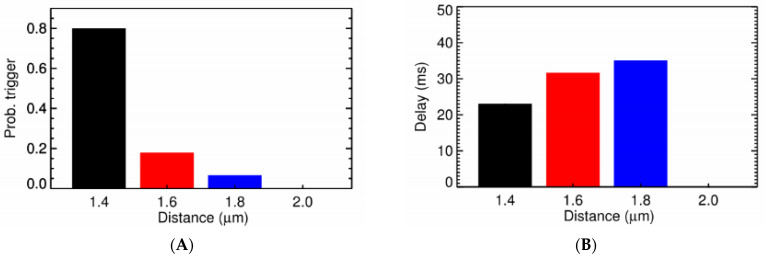
**(A)** The P_o,trigger_ of Ca^2+^ release from 9 activated CRUs on one Z-line on the CRU at different distances. (**B**) The time delay for the activation at different distances.

**Figure 11:**
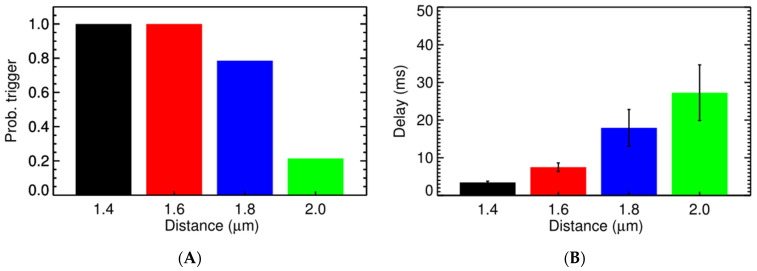
**(A)** The P_o,trigger_ of Ca^2+^ release from 9 activated CRUs on one Z-line, with 1 intermediate RyR2 cluster in the middle, on the CRU at the next Z-line of different distance. (**B**) The time delay for the activation at different distances.

We calculated the propagation velocity as shown in the case of spark-induced spark, when the source of Ca^2+^ is from a single release site, the velocity is 30–45 µm/s. Using 9 activated CRUs, the wave velocity in the simulation setting, as shown in Figure 10, is 63.6 µm/s at 1.4 µm apart and then reduced to 48.4 µm/s at 1.8 µm apart. Another simulation series was created where one intermediate cluster of RyR2s was added, Figure 11. When an intermediate RyR2 cluster was added at 0.6 µm apart, not only the probability for trigger P_o,trigger_ increased but also the wave speed was 74 µm/s (for Z-line 2.0 µm apart) and 100 µm/s (for Z-line 1.8 µm apart). This suggested that the wave velocity is strongly dependent on the mass of calcium release, i.e., the number of activated CRUs on one Z-line and the distance to the CRU in the next Z-line and suggests an important role of intermediate RyR2 clusters.

**Figure 12:**
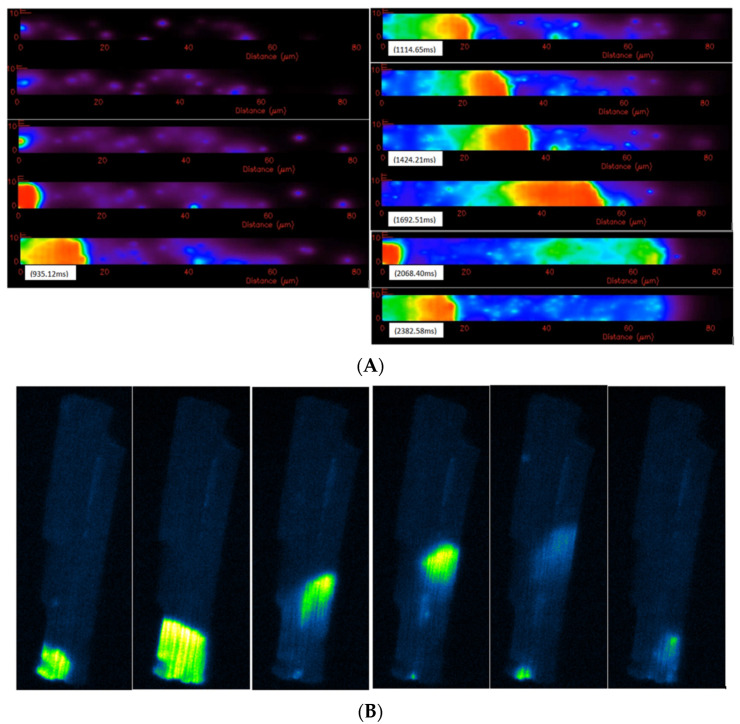
**(A**) Calcium overload ([Ca]_nsr_ = 1.7 mM, [Ca]_i_ = 0.15 μM), this computational model of the rat ventricular myocyte can reproduce a repetitive sustained calcium wave which typically initiates at one end of the cell. The initiation site typically occurs where release sites are closer together or at a boundary. (**B**) Calcium waves in the rat ventricular myocyte under [Ca]_o_ = 5 mM overload experimental conditions, the repetitive waves occur at a particular site for each cell. This suggests that there are more density of release sites surrounding the region that allows mass calcium release high enough to trigger the wave. Some waves can sustain to the next end, while some decay and stop in between, which suggests a stochastic nature of the waves (personal communication from Brian Hagen).

**Figure 13:**
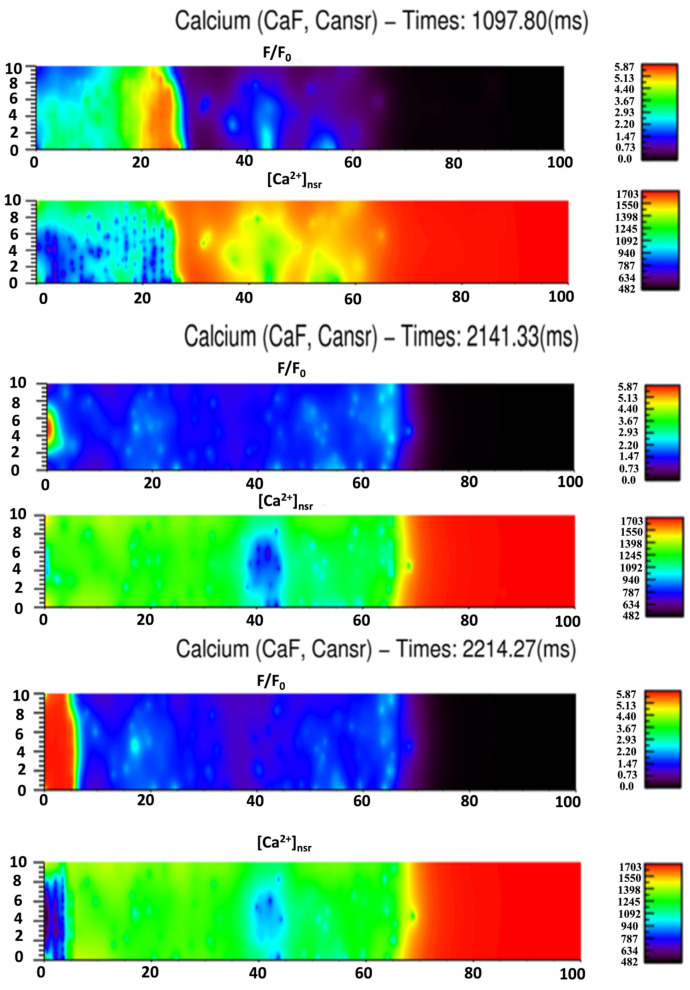
Given the initial [Ca]_nsr_ = 1.7 mM, to derive the triggering of the wave, the simulation suggested that an overload of 1.5 mM is enough to trigger the repetitive calcium waves.

The results presented strengthen the positive amplitude-velocity relationship that has been suggested by previous experiments [95,96]. The estimated wave velocities vary significantly between different studies. The early estimation of wave velocity in cardiac myocytes was 100 µm/s [97]. Recently, by testing different diffusion constants of calcium, [98] estimated the value to be around 78 µm/s. Experimental data by [92] in rabbit ventricular myocytes suggested the range 102.2 ± 4.19 µm/s. In other experiments for rats, the authors have showed that the wave speed strongly depends on the amount of calcium overload and temperature [99,100]. Using [Ca^2+^]_o_ from 2 mM to 15 mM, the wave speed increases from 33 µm/s to 87 µm/s; while at [Ca^2+^] = 3 mM, the wave speed was 33 µm/s at 23 °C and 74 ± 19 µm/s at 30 °C. Another experiment in rats confirmed the diversity in spatiotemporal properties of Ca^2+^ waves, modulated by the Ca^2+^-loading state [101] with different wave velocities: 84 ± 16 µm/s and 116 ± 29.4 µm/s. This is in agreement with our results, that the wave speed varies with the mass calcium release that elevates basal levels of [Ca^2+^]_i_. In addition, we showed the important role of off-Z-lines CRUs in wave propagation. A whole-cell simulation of Ca^2+^ wave is shown in Figure 12A. The model produced repetitive waves similar to Ca^2+^ waves in experiments under Ca^2+^ overload conditions, as shown in Figure 12B (Supplemental Videos S1 and S2). Figure 13 shows the simulated fluorescence and network SR Ca^2+^ changes accompanying the Ca^2+^ waves at three example times during the simulation. Note that the fall in the network SR calcium occurs slightly behind the Ca^2+^ wave front as diffusion in the myoplasm is the key driver of wave propagation due to CICR. It is important to note that the irregular spacing of the release sites was essential for wave propagation because in the model with uniform site placement, waves did not propagate beyond a few sarcomeres.

**Figure 14:**
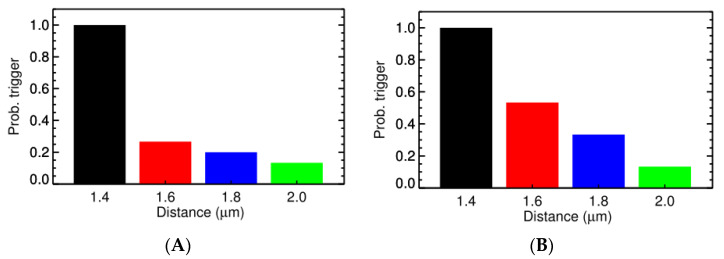
Effect of SERCA on the probability of a Ca^2+^ triggering a Ca^2+^ wave. (**A**) uniform SERCA distribution; (**B**) 90% reduction in SERCA flux.

The role of SERCA in the spark-induced Ca^2+^ wave was tested, as shown in Figure 14. Compared to Figure 10A, with uniform distribution of SERCA along the longitudinal direction (Figure 14A), Ca^2+^ waves can propagate farther because less Ca^2+^ will be sequestered at the release site. When SERCA is reduced, the waves can propagate farther because there is more Ca^2+^ available for diffusion (Figure 14B). This is in agreement with a previous study that found SERCA inhibition should enhance the wave velocity [102]. Given the fact the cytosolic calcium diffuses faster than calcium in the SR, the inhibition of SERCA should increase the diffusion of calcium to the next release site to trigger the opening of RyR2 there. After applying UV-flash photolysis of ‘caged’ Nmoc-DBHQ or the blocking of SERCA affecting the activation of CRUs (a precursor of the SERCA-inhibitor DBHQ [103]), Kelly and co-workers found a decrease in Ca^2+^ wave velocity. The authors suggested that calcium overloaded at one CRU, due to the activity of the SERCA pump, leads to RyR2 sensitization ahead of the cytosolic Ca^2+^ wave, which explains the reason why blocking SERCA decreases the Ca^2+^ wave [104]. However, this does not follow the CICR mechanism. Based on a computational study, Ramay and co-workers concluded that this ‘sensitization’ only occurs at a slow diffusion of SR Ca^2+^ [27]. However, this slow diffusion was not found in other studies [74]. The activation of one CRU, during a Ca^2+^ wave, depends on the Ca^2+^ released from CRUs, not only from the adjacent Z-line, which in turn depends on the distance, but also from activated CRUs on the same Z-line. Thus, one possible explanation for this when SERCA is blocked is when they measure the velocity, the CRU distance is longer for the Ca^2+^ to reach and the blocking of SERCA reduces the activated CRUs on the same Z-line, which in turn reduce diffusing Ca^2+^ to activate the release site.

## 4. Discussion

A 3D spatiotemporal model has been used in this investigation to study spark-induced sparks and spark-induced Ca^2+^ waves. The model was developed using available data for rat ventricular myocytes from the literature. This model is superior to other models in many ways. The first is that this realistic spark model has been tested to reproduce proper pump/leak balance in rat ventricular myocytes [84]. The second is the detailed 3D spatiotemporal representation of the cell at the resolution of 200 nm. The third is the realistic representation of different ionic currents on the SL, SR membranes, as well as the non-uniform distribution of the endogenous buffers in the cells. The model’s results are in agreement with other studies that the Ca^2+^ wave velocity varies upon the condition of the setting, in particular the loading of Ca^2+^. The important role of intermediate clusters has been shown in the model as well, which has not been investigated before. This is in agreement with experimental data showing that T-tubules are a far more complicated structure that branches not only transversally but also longitudinally [47]. Moreover, the role of satellite clusters was also tested, which implied a heterogeneity in RyR2 cluster arrangements. The widely used assumption is that a single connected RyR2 cluster forms a planar array for each release site. However, the experimental data have showed RyR2 arrangement is more heterogeneous and that functional release sites can be the result of one main RyR2 cluster and a few smaller sized RyR2 clusters at a distance of 100–200 nm apart [77,89,90,91]. By using a model with one central cluster with 2 satellite clusters, the model was able to reproduce the proper FWHM of an experimentally observed Ca^2+^ spark.

Using the local control model, the results showed how SR Ca^2+^ can induce Ca^2+^ waves based on stochastic gating of RyR2 channels. In the current model, we suggest that an activation of 7–9 CRUs is required to trigger a wave from one Z-line to another. However, the probability of activation depends on the distance. There are two factors that may contribute to this increase in spark-induced spark activity leading to wave propagation. The first is the amplification of the Ca^2+^ signal by the opening of RyR2 channels from these satellite clusters. In addition, it has been suggested that clusters of smaller sizes tend to open more often due to the weak allosteric coupling between the channels [84,105]. Thus, we suggested that the high opening activity of these satellites is the second factor that contribute to this rising in spark-induced-spark phenomenon. The overal effect is quite important as we can now see that a CRU can activate another one at a farther distance, forming the so-called ‘macrosparks’ that have been observed in other experiments [2,14,93,106]. The diffusion constant of ATP as well as CaATP is significant, 168 μm^2^/s [82]. The space consumed by the T-tubule has not been considered, i.e., it was assumed to be zero. Adding these to the model may help to boost Ca^2+^ diffusion as a fraction of space is now consumed by the T-tubule and the diffusion of calcium is facilitated by Ca-ATP. Thus, it may help to spread Ca^2+^, which makes wave initiation and propagation even easier. Other factors that may affect Ca^2+^ wave or arrhythmias that have not been included in the model are the presence of mitochondria and the nucleus [107,108,109].

The T-tubule structure is complex with recent studies showing it varies for different myocytes in the heart [110]. Atrial myocytes have a lower T-tubule density than ventricular myocytes [111]. Remodeling of the T-tubular network has been observed during disease [112]. Furthermore, during disease there is also remodeling of the T-tubular network post-myocardial infarction and is present during cardiac hypertrophy and heart failure [56,113]. In fact, remodeling of the T-tubular network has been shown to disrupt β-adrenergic signaling during heart failure [114]. Computational modeling has been used to help understand the implications of T-tubule remodeling on cardiac calcium dynamics. Wagner et al. demonstrated, with computational modeling, that the T-tubule remodeling causing reduction SR and T-tubule proximity post myocardial infarction led to delayed subcellular Ca^2+^ release and action potential prolongation [113]. Other work by Song et al., using a spatial computational model, showed that Ca^2+^ alternans and Ca^2+^ wave-mediated arrhythmias increased when the percentage of orphaned Ca^2+^ release sites was in an intermediate range, but reduced in myocytes exhibiting either a well-organized T-tubule network or low T-tubule density [115]. The model presented here demonstrated the importance of the T-tubule location and non-uniformity of placement for the generation of Ca^2+^ waves because close proximity of some Ca^2+^ release sites is needed for the Ca^2+^ wave nucleation and propagation, consistent with these previous findings.

Previous studies have explored the distance between release sites as being an important factor for Ca^2+^ wave propagation. Izu and co-workers were able to achieve propagation between z-disks in a cardiac myocyte, but required large Ca^2+^ sparks to achieve this [26]. Nivala and co-workers demonstrated the importance of distance in a simplified model of Ca^2+^ release [116]. In a simulation site, Coleman and co-workers observed that the distance between release sites was an important factor in Ca^2+^ propagation [117].

The importance of rogue RyR2s in SR Ca^2+^ leaks has been previously described [84,105]. They have also been suggested to be critical for Ca^2+^ wave propagation [118]. However, in this work Ca^2+^ waves could propagate between sites at normal SR [Ca^2+^]. A simulation study by Chen and co-workers observed that rogue ryanodine receptors were needed to get propagation between release sites [119]. However, a single spark could not initiate a Ca^2+^ wave. Four adjacent release sites had to be triggered to initiate a wave. This condition is not required in our model. It could be because the modeling work presented here paid careful attention to achieving accurate Ca^2+^ spark dynamics and FWHM.

To understand Ca^2+^ wave propagation it is essential to capture correct Ca^2+^ spark dynamics and FWHM. Experimentally observed FWHM of recorded Ca^2+^ sparks is about 1.8–2.2 µm. Previous computational models have not been able to produce realistic FWHM, only reaching up to ~1.2 µm [15,16,17,18,19,20,21,120]. Izu and co-workers developed a 3D model with spherical geometry using FASCIMILE (AEA Technologies, Harwell, UK) for studying Ca^2+^ sparks. In their model, in order to reproduce FWHM ~2 µm, they created a supercluster with 4 CRUs at 0.4 µm apart and a very large release of Ca^2+^ was assumed, i.e., from 2 pA to 5–10 pA for each CRU [17]. A recent 3D model developed using FASCIMILE by Kong and co-workers also produced FWHM of 1.2 μm [22]. The computational model presented here was able to simulate realistic FWHM of 1.85 µm which is sufficient to cover the sarcomere with elevated Ca^2+^ and ensure contraction.

The current model hasn’t incorporated the different oligomers of CSQ, thus the total free SR [Ca^2+^] during a calcium wave has not been investigated. However, we were able to produce, for the first time, a repetitive Ca^2+^ wave at [Ca^2+^]_SR_ = 1.7 mM and [Ca^2+^]_myo_ = 0.0156 μM. The simulation showed that the local high elevation of cytosolic calcium, which was significantly underestimated using the back-calculation method, may play an important role in regulating the cellular signals. Lastly, the non-uniform placement of CRU is important for calcium wave initiation and propagation under calcium overload.

Keller and co-workers [104] proposed the concept of RyR2 ‘sensitization’ as a possibility for decreasing Ca^2+^ wave velocity. We did the test by (1) reducing SERCA during Ca^2+^ waves, (2) by using a different SR Ca^2+^ diffusion constant, however the result follows CICR. As we pointed out, the Ca^2+^ velocity depends not only on the CRU distance but also the role of intermediate CRU’s. Thus, it is possible that the increase/decrease in Ca^2+^ wave velocity is the result of the presence of an intermediate RyR2 cluster or a longer CRU distance. Another possible explanation is that with SERCA blocking, the number of activated CRUs on one Z-line can be smaller, thus reducing the mass Ca^2+^ release, which in turn reduces the Ca^2+^ wave velocity. We thus reject the hypothesis that the Ca^2+^ diffusion constant is higher on the SR side.

Using the Ca^2+^-sensitive RyR2’s compatible with experimental measurements, the model has successfully explained the difference in wave speed, the mechanism and conditions at which Ca^2+^ waves are initiated. In an earlier 2D model, Ca^2+^-bound fluorescence had not declined to the baseline level after the wave [26]. The model, for the first time, is able to reproduce repetitive Ca^2+^ waves, without any change to RyR2 channel Ca^2+^-sensitivity and the SERCA pump. The model is the first of its kind at this level of detail and promises to be able to provide a further understanding of different pathophysiological conditions, such as T-tubule remodeling or diverse channel mutations.

## 5. Conclusions

Our spatiotemporal model of Ca^2+^ dynamics simulates realistic spark frequency, dynamics, and FWHM. This is the first model to produce realistic FWHM with physiologically realistic parameters. The model suggests that the robustness of Ca^2+^ wave propagation depends on Ca^2+^ release site placement, the density of RyR2 clusters, the presence of non-junctional or rogue RyR2 channels, and the level of the SR Ca^2+^ load. The model shows that repetitive Ca^2+^ waves can be produced under Ca^2+^ overload conditions, similar to those observed in recorded experiments.

## Figures and Tables

**Table 1:** Percentage of Ca^2+^ sparks resulting in a Ca^2+^ wave.

	CRU Dist.	0.6	0.8	1.0
RyR2 Sense				
[Ca^2+^]_sr,max_ = 1.13 mM		93.22%	1.27%	0.8%
[Ca^2+^]_sr,max_ = 1.3 mM		95.48%	1.55%	1%

P_o,trigger_ when [Ca^2+^]_myo_ = 0.156 µM, and [Ca^2+^]_sr_ = 1.3 mM. The latter case assumes lumenal Ca^2+^ sensitivity saturate at 1.13 mM.

## Data Availability

Model codes are publicly available at the Mason Archival Repository Service (MARS) at the following link: Available online: http://mars.gmu.edu/handle/1920/12166 (accessed on 14 December 2021).

## Supplementary Materials

The following are available online at https://www.mdpi.com/article/10.3390/membranes11120989/s1, Video S1: Experimental Calcium Waves) and Video S2: Simulated Calcium Waves.

## Appendix A

### Appendix A.1. Parameters

**Table A1:** Parameters in the model.

Parameter	Definition	Value
V_cell_	Cell volume	24.96 (pL)
V_myo_	Myoplasmic volume	50% × V_cell_
V_nsr_	Network SR volume	3.2% × V_cell_
V_jsr_	Junctional SR volume	0.5% × V_cell_
Dyad_height	The distance between jSR and T-tubule	12 nm
Couplon_size	The size of the subspace	300 nm
X_len	Cell dimension	120 µm
Y_len	Cell dimension	20.8 µm
Z_len	Cell dimension	10.0 µm
D_myo_(x,y,z)	Free cytosolic calcium diffusion	270 µm^2^/s
D_nsr_(x,y,z)	Free SR calcium diffusion	60 µm^2^/s
D_dye_(x,y,z)	Fluorescence and calcium-bound fluorescence diffusion	90 µm^2^/s
F_T_	Total fluorescence (Fluo-3)	50 µM
k_on_	Binding constant for fluorescence	80 µM^−1^.s^−1^
k_off_	Unbinding constant for fluorescence	72 s^−1^
E_cc_	Change in free energy between closed RyR2 pairs	−0.872 k_B_T
E_oo_	Change in free energy between open RyR2 pairs	−1.15 k_B_T
k_B_	Boltzmann constant	1.381 × 10^−23^ J/K
T	Temperature	295.15 K
[K^+^]_o_	Extracellular potassium concentration	5.4 mM
[Na^+^]_o_	Extracellular sodium concentration	140 mM
[Ca^2+^]_o_	Extracellular calcium concentration	1.8 mM
[K^+^]_i_	Cytosolic potassium concentration	143.72 mM
[Na^+^]_i_	Cytosolic sodium concentration	10.2 mM
[Ca^2+^]_i_	Cytosolic calcium concentration	0.096 µM
[Ca^2+^]_sr_	SR calcium concentration	1.02 mM
B_myoT_	Total myoplasmic buffer concentration	3.703026 × 10^−2^ µM
K_m,myo_	Disassociation constant of myoplasm buffer	1.1900 µM
[Trpn]	Troponin Ca^2+^ binding sites concentration (k^+^ = 2.37 µM^−1^.s^−1^, k^−^ = 0.032 s^−1^)	140 µM
[Calm]	The dynamics buffers with K_d_ = 2.38 µM (k^+^ = 30 µM^−1^.s^−1^, k^−^ = 71.4 s^−1^)	24 µM
[B]_SL_	The SL buffer (k^+^ = 115 µM^−1^.s^−1^, k^−^ = 1000 s^−1^)	750 µM
[B]_SR_	The SR buffer with Kd=0.86µM (k^+^ = 115 µM^−1^.s^−1^, k^−^ = 100 s^−1^)	47 µM
A_pump_	Concentration of SERCA pump	300 µM
K_p,myo_	The binding affinity of cytosolic calcium to SERCA	900 µM
K_p,nsr_	The binding affinity of SR calcium to SERCA	2150 µM
v^T^_refill_	Total refill rate for 20,000 CRUs	3 (1/s/(L-cyt))
v^T^_efflux_	Total efflux rate for 20,000 CRUs	120 (1/s/(L-cyt))
i_ryr_	Single channel RyR2 current	0.2 (pA)
g_bCa_	Background Ca^2+^ conductance	2.9 × 10^−4^ (mS/µF)
g_bNa_	Background Na^+^ conductance	1.066 × 10^−4^ (mS/µF)
g_bK_	Background K^+^ conductance	0 (mS/µF)
g_Na_	Na^+^ conductance	13 (mS/µF)
g_K1_	K_1_ conductance	0.2 (mS/µF)
g_Kss_	K_ss_ conductance	4.21 × 10^−2^ (mS/µF)
g_Ktof_	K_tof_ conductance	0.0798 (mS/µF)
g_Ktos_	K_tos_ conductance	6.29 × 10^−2^ (mS/µF)
A_m_	Cell surface area	
I_pmca_	Maximum PMCA current density	0.10 (µA/µF)
I_ncx_	Maximum NCX current density	750 (µA/µF)
I_NaK_	Maximum NaK current density	0.88 (µA/µF)
eta_RyR	Hill coefficient of Ca^2+^-dependent in RyR	2.2
eta_LCC	Hill coefficient of Ca^2+^-dependent in LCC	2.0
E_j_	Coupling strength energy (for 49 RyRs)	0.0714
Ecc	Coupling energy between 2 closed channels	−0.78 (k_B_T)
Eoo	Coupling energy between 2 open channels	−1.26 (k_B_T)
