# Supplementary material for: A Stochastic Spatiotemporal Model of Rat Ventricular Myocyte Calcium Dynamics Demonstrated Necessary Features for Calcium Wave Propagation
Source: Membranes (Basel). 2021 Dec 18;11(12):989. doi: 10.3390/membranes11120989 (PMC8706945; doi:10.3390/membranes11120989)
Supplement: Supplementary file 1 [file membranes-11-00989-s001.zip › Supplementary Caption.pdf]

Experimental (S1 – Experimental Calcium Waves) and simulated (S2 – Simulated Calcium Waves) calcium waves under calcium overload conditions.
